# Quantifying the effects of cold waves on carbon monoxide poisoning: A time-stratified case-crossover study in Jinan, China

**DOI:** 10.3389/fpubh.2023.1050256

**Published:** 2023-04-18

**Authors:** Jinli Wei, Aifeng Ren, Yingjian Zhang, Yuanrong Yin, Nan Chu, Yiwen Ma, Jipei Du, Liangliang Cui, Chengchao Zhou

**Affiliations:** ^1^Centre for Health Management and Policy Research, School of Public Health, Cheeloo. College of Medicine, Shandong University, Jinan, China; ^2^Jinan Medical Emergency Center, Jinan, China; ^3^Jinan Municipal Center for Disease Control and Prevention, Jinan Municipal Center for Disease Control and Prevention Affiliated to Shandong University, Jinan, China; ^4^NHC Key Lab of Health Economics and Policy Research, Shandong University, Jinan, China

**Keywords:** cold wave, carbon monoxide poisoning, weather warnings, time-stratified case-crossover, climate effects on CO poisoning

## Abstract

**Background:**

Previous studies have shown that carbon monoxide (CO) poisoning occurs mostly in winter and is associated with severe cold weather (e.g., ice storms, temperature drops). However, according to previous studies, the impact of low temperature on health has a delayed effect, and the existing research cannot fully reveal the delayed effect of cold waves on CO poisoning.

**Objectives:**

The purpose of this study is to analyze the temporal distribution of CO poisoning in Jinan and to explore the acute effect of cold waves on CO poisoning.

**Methods:**

We collected emergency call data for CO poisoning in Jinan from 2013 to 2020 and used a time-stratified case-crossover design combined with a conditional logistic regression model to evaluate the impact of the cold wave day and lag 0–8 days on CO poisoning. In addition, 10 definitions of a cold wave were considered to evaluate the impact of different temperature thresholds and durations.

**Results:**

During the study period, a total of 1,387 cases of CO poisoning in Jinan used the emergency call system, and more than 85% occurred in cold months. Our findings suggest that cold waves are associated with an increased risk of CO poisoning in Jinan. When P01, P05, and P10 (P01, P05, and P10 refer to the 1st, 5th, and 10th percentiles of the lowest temperature, respectively) were used as temperature thresholds for cold waves, the most significant effects (the maximum OR value, which refers to the risk of CO poisoning on cold wave days compared to other days) were 2.53 (95% CI:1.54, 4.16), 2.06 (95% CI:1.57, 2.7), and 1.49 (95% CI:1.27, 1.74), respectively.

**Conclusion:**

Cold waves are associated with an increased risk of CO poisoning, and the risk increases with lower temperature thresholds and longer cold wave durations. Cold wave warnings should be issued and corresponding protective policies should be formulated to reduce the potential risk of CO poisoning.

## 1. Introduction

Existing research shows that extreme temperatures can lead to increased incidence and mortality of disease, which is a serious threat to people’s health (1, 2). A cold wave is a form of extreme weather that happens frequently in the winter and is characterized by widespread severe cold temperatures, high winds, rain, and snow. In the past few years, frequent cold waves have had a non-negligible impact on human health, which has led to increasing public concern about cold waves. China is located in the northern half of the Eastern Hemisphere, east of Eurasia, and the west coast of the Pacific Ocean, and cold waves often occur (3). Cold waves seriously affect public transportation and production activities, resulting in a huge economic loss (4, 5). In November 2015, large-scale cooling occurred in northern China, and the lowest temperature in many places fell below the record for the lowest November temperature since 1961 (6). Therefore, it is necessary to further study the impact of cold waves on health in China.

Carbon monoxide (CO) poisoning is a major global public health event and is one of the most common types of fatal poisoning in many countries (7). Severe CO poisoning can lead to confusion, respiratory depression, and even death (8). According to the statistics, the global cumulative incidence of CO poisoning was approximately 137 cases/1 million people, and the mortality rate was approximately 4.6 people/1 million people in 2017 (9). In 2019, there were approximately 970,000 cases of accidental CO poisoning worldwide, with a mortality rate of approximately 4.2% (10). The sequelae of more than 35% of cases manifest in the nervous system (11), which significantly increases the burden on families and society (12). In Western countries, cases of CO poisoning have been well reported, and researchers have mainly divided them into accidental death and suicide (13–16). In the United States alone, approximately 2,700 people die from CO poisoning a year (17). However, studies on CO poisoning in mainland China are relatively lacking.

In China, CO poisoning is the third biggest cause of deaths in families, after traffic accidents and production accidents (People’s Government of Laiwu District, Jinan City). Approximately 70,000 people are poisoned by CO due to the incomplete combustion of gas every year, and it is the second most common cause of poisoning in adults in China (18); CO poisoning is still a non-negligible problem in China. In the first quarter of 2022 alone, a total of 278 gas accidents on the Chinese mainland were reported.^1^ Exposure to CO is not easily identified, so identifying factors that contribute to CO poisoning is critical for developing prevention and control strategies.

Several studies have shown that CO poisoning events are related to cold weather, especially extreme weather such as cold waves, blizzards, and ice storms. A meta-analysis in Iran showed that CO poisoning incidents peaked in December and trended downward in March the following year (19). In extreme cold weather, CO poisoning events are not uncommon; in the 2002 North Carolina and 2009 Kentucky ice storms, 50 and 144 cases of poisoning were observed, respectively (20, 21). These studies show a correlation between CO poisoning and low temperatures, but there are also studies showing that the health effects of low temperatures have a lag (22, 23), that is, the health effects of low temperatures persist for a period of time. In addition, no one has specifically studied the association between CO poisoning and different definitions of a cold wave so far. These studies cannot accurately determine the time range of the influence of cold waves on CO poisoning, so it is necessary to carry out relevant studies to further determine the effect of cold waves on CO poisoning.

Research suggests that the extreme cold in the 20th century may not be lower or shorter than recent averages in many places (24). China has experienced many cold waves, especially in northern China. In Jinan, the temperature is low in winter, and cold waves and CO poisoning are prone to occur. From 2007 to 2017, a total of 4,403 non-occupational CO poisoning cases were reported, with 161 deaths and a case fatality rate of 3.5% (25). However, little is known about the risk distribution of CO poisoning following cold wave exposure. To bridge this gap, this study aims to use a time-stratified case-crossover approach to quantitatively assess the relationship between cold waves and CO poisoning in the general population in Jinan City. The specific objectives are to (1) clarify the distribution of CO poisoning and meteorological factors in Jinan City, as well as the long-term trends; and (2) compare the impact of various definitions of a cold wave on CO poisoning, including the single-day lag effect and cumulative effect, so as to provide evidence for issuing different levels of warnings.

## 2. Materials and methods

### 2.1. Study site and study period

Jinan City (latitude 36°40′ north, longitude 117°00′ east) is the capital of Shandong Province. It is located in the central and western parts of Shandong Province, eastern China. Jinan City covers an area of 8,177 km^2^. In 2020, the resident population of Jinan was approximately 9.2 million (Shandong Provincial Statistical Yearbook 2020). Jinan has a monsoon climate. The four seasons are distinct: the spring is dry and less rainy, the summer is warm and rainy, the autumn is cool and dry, and the winter is cold and snowy. We limited the study period to January 1, 2013 to December 31, 2020.

### 2.2. Data collection

#### 2.2.1. Data of CO poisoning event

The Jinan Emergency Center records all emergency calls in Jinan. We obtained ambulance call data from Jinan Emergency Call Center from January 1^st^ 2013 to December 31^st^ 2020, including call time, call reason, call age, gender, and call address. According to the “call reason,” we extracted data on calls for help due to carbon monoxide poisoning for our research. Cases with residential addresses outside Jinan were excluded.

#### 2.2.2. Meteorological and air pollution data

The daily average temperature (T_average_, °C), daily maximum temperature (T_max_, °C), daily minimum temperature (T_min_, °C), daily average pressure (Pressure, kPa), average relative humidity (RH, %), and wind speed (wind, m/s) were observed at Jinan Meteorological Station (China Meteorological Administration ID54823). These data came from the National Meteorological Information Center of China.^2^

The daily concentration of pollutants is provided by Jinan Environmental Monitoring Center, including the maximum ambient ozone concentration over an 8-h period.

(O_3_, μg/m^3^), daily mean particulate matter with aerodynamic diameter < 2.5 μm (PM2.5, μg/m^3^), daily mean particulate matter with aerodynamic diameter < 10 μm (PM10, μg/m^3^), average daily concentration of sulfur dioxide (SO_2_, μg/m^3^), average daily concentration of nitrogen dioxide (NO_2_, μg/m^3^), and average daily concentration of carbon monoxide in the environment (CO, μg/m^3^). Previous studies have shown that air pollutants can have negative effects on the human body (26, 27), so we used air pollutant concentrations to test the stability of our model.

### 2.3. Study design and statistical analysis

#### 2.3.1. Cold wave definition

The definition of a cold wave is varied in previous studies; in most cases, a cold wave is defined as a few days when the temperature is below a certain threshold (28, 29). In this study, based on the actual situation of Jinan City, we used the 1st, 5th, and 10th percentiles of daily minimum temperature as three thresholds (for P01, P05, and P10, the temperature is −8.9, −5.0, and −2.5°C, respectively), combined with four durations (≥2, ≥3, ≥4, and ≥5 days), resulting in a total of 12 definitions, denoted Pi(j), where “i “means that the temperature threshold is the i-th percentile of the daily minimum temperature and “j “means the duration is not less than j days. The threshold temperature must be exceeded on each day of the cold wave (for example, P05(4) represents the value of the daily minimum temperature below the 5th percentile of the daily minimum temperature for at least 4 consecutive days). When defined as P01(4) and P01(5), the frequency of cold waves is too small, at 2 and 0 times, respectively. Therefore, we established 10 cold wave definitions.

#### 2.3.2. Cold wave impact

First, we performed a descriptive analysis of daily meteorological data and CO poisoning cases from 2013 to 2020 and observed the changing trend of CO poisoning during the study period. Then, to reduce the multicollinearity of the model, we performed a nonparametric Spearman correlation analysis on the meteorological variables. If the variables are highly correlated (|r| > 0.7) (30), only one variable is left and the rest will not be included in the final model.

We explored the acute effects of cold waves on CO poisoning events. A time-stratified case-crossover design (31) combined with conditional logistic regression models was used to examine the association between cold waves and the risk of CO poisoning events. Since the case-crossover design compares events (cases) to themselves at different points in time, it adjusts for factors that do not vary within individuals over short periods. In this study, matched sets consisting of the event day (the day of CO poisoning events occurring) and control days were selected on the same day of the week in the same month and year as the event day (i.e., 3–4 referent days per event). This time-stratified approach to referent selection has been shown to result in unbiased conditional logistic regression estimates in case-crossover studies. The model is as follows:


$$\log\;\left(E\;\left[Yt\right]\right)=\alpha +\beta X+RH+\mathrm{wind}+\mathrm{holiday}+\mathrm{stratum}$$

where t refers to the day, Yt refers to the number of people with CO poisoning, Log (E[Yt]) refers to the risk function, α is the intercept, β refers to the coefficient vector, and X refers to the cold wave. In the analysis, we simulated the cold wave with binary variables. The cold wave day was recorded as 1, otherwise, it was recorded as 0; stratum is a variable that matches by year, month, and week.

After an exploratory analysis, and referring to previous studies (32), we used a lag time of 8 days to analyze the short-term effects of cold waves. Different exposure lag structures were evaluated for cold waves in separate models: Lag0 (the same day as the CO poisoning events occurred), Lag1 (the day before the events occurred), and so on until Lag8 (8 days before the events occurred); Lag01 (cumulative effect of a 1-day lag), up to Lag08 (cumulative effect of an 8-day lag). In order to control the confounding effects of other meteorology, daily relative humidity, and wind speed were added to the analysis. The results are expressed as OR (odds ratio) with a 95% CI (95% confidence interval).

As for the sensitivity analysis, we added PM_2.5_, PM_10_, NO_2_, CO, O_3_, and SO_2_ into the main model and then compared it with the main model results.

The statistical analyses were conducted using the R statistical software (version 3.3.2). *p*-value < 0.05 is considered statistical significance.

## 3. Results

Table 1 shows the basic situation of daily CO poisoning quantity, meteorological factors, and air pollutants in Jinan City during the study period. During the study period, a total of 1,387 cases of CO poisoning were recorded by the Jinan Emergency Call Center, and the number of emergency calls for CO poisoning varied from 0 to 12 cases per day. More than 85% of CO poisoning cases occurred in cold months (November, December, January, February, and March). During the study period, the number of people poisoned by CO showed an overall decreasing trend, and the decrease was more obvious after 2018. Moreover, the number of poisonings in 2018–2020 was only 19.4% of the total number of poisonings in the study period (Supplementary material S1). The average value of *T*_min_ in the same period was 11.3°C, and the average values of wind, RH, and pressure were 2.37 m/s, 55.9%, and 996.7 kPa, respectively. The average concentrations of PM2.5, PM10, SO_2_, NO_2_, CO, and O_3_ were 72.08, 138.4, 41.76, 46.79, 1,158, and 109.6 μg/m^3^, respectively.

**Table 1:** Descriptive characteristics of daily CO events and meteorological conditions from 2013 to 2020, Jinan City.

Variable	Mean ± SD	Min	P25	P50	P75	Max
CO events	0 ± 1	0	0	0	1	12
Meteorological factors						
*T*_average_ (°C)	15.36 ± 10.35	−12.40	6.10	16.70	24.50	33.80
*T*_min_ (°C)	11.3 ± 10.08	−17.0	2.3	12.4	20.2	30.8
*T*_max_ (°C)	20.4 ± 10.66	−7.1	11.3	22.1	29.8	39.9
Wind (m/s)	2.37 ± 1.04	0.200	1.625	2.100	2.800	8.400
RH (%)	55.59 ± 18.97	15.00	40.00	55.00	70.00	100
Pressure (hPa)	996.7 ± 9.3	975.1	988.6	996.9	1004.0	1021.8
Atmospheric pollutant						
PM2.5 (μg/m^3^)	72.08	3	39	59	90	443
PM10 (μg/m^3^)	138.4	5	87	123	170	693
SO_2_ (μg/m^3^)	41.76	5	15	28	51	429
NO_2_ (μg/m^3^)	46.79	9	32	44	57	165
CO (μg/m^3^)	1,158	363	790	1,011	1,349	6,555
O_3_ (μg/m^3^)	109.6	5	62	102	153	268

Table 2 shows the number of cold wave counts, cold wave days, and the number of CO poisoning events during the cold wave for 10 different definitions. The higher the temperature threshold and the shorter the duration, the more cold waves occur. The number of CO poisoning incidents during the period also increased. This may be because there are fewer extreme cold days and fewer consecutive extreme cold days. So the higher the threshold of the definition of a cold wave, the more days are included in the definition, that is, the more “cold wave days” there are.

**Table 2:** Number of cold waves, days of cold waves, and CO events during cold waves 2013–2020, Jinan.

Definition	Temperature (°C)	Duration (days)	Cold wave times	Cold wave days	Event
P01(2)	≤1th	≥2	8	22	49
P01(3)	≤1th	≥3	4	13	31
P01(4)*	≤1th	≥4	2	8	18
P01(5)*	≤1th	≥5	1	0	0
P05(2)	≤5th	≥2	36	117	168
P05(3)	≤5th	≥3	21	87	139
P05(4)	≤5th	≥4	12	60	114
P05(5)	≤5th	≥5	5	32	75
P10(2)	≤10th	≥2	60	258	346
P10(3)	≤10th	≥3	40	218	292
P10(4)	≤10th	≥4	28	182	245
P10(5)	≤10th	≥5	19	146	201

* Indicates that the number of cold waves that occurred was too low to be included in the study.

**Figure 1:**
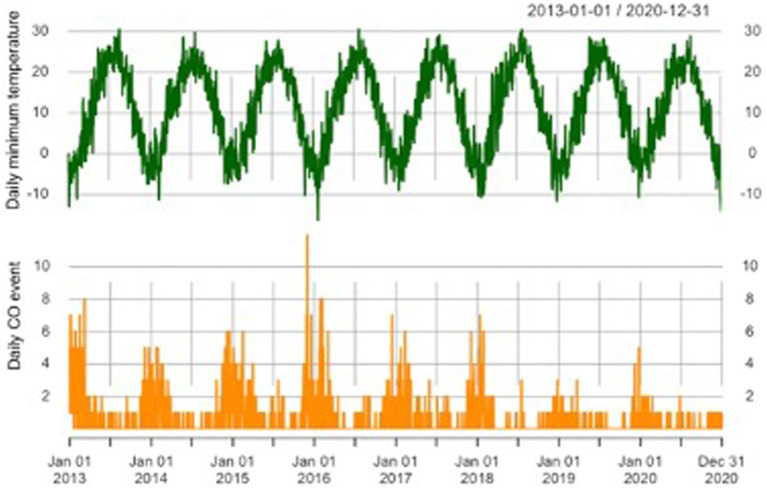
The time series distribution of daily minimum air temperature and CO poisoning events in Jinan City.

Figure 1 shows the time series distribution of the number of CO poisoning incidents and daily minimum temperature in Jinan from 2013 to 2020. Both CO poisoning events and daily minimum temperatures are cyclical. The nadir of the daily minimum temperature and the peak of CO poisoning events occurred around January each year, suggesting a possible link between the two.

**Figure 2:**
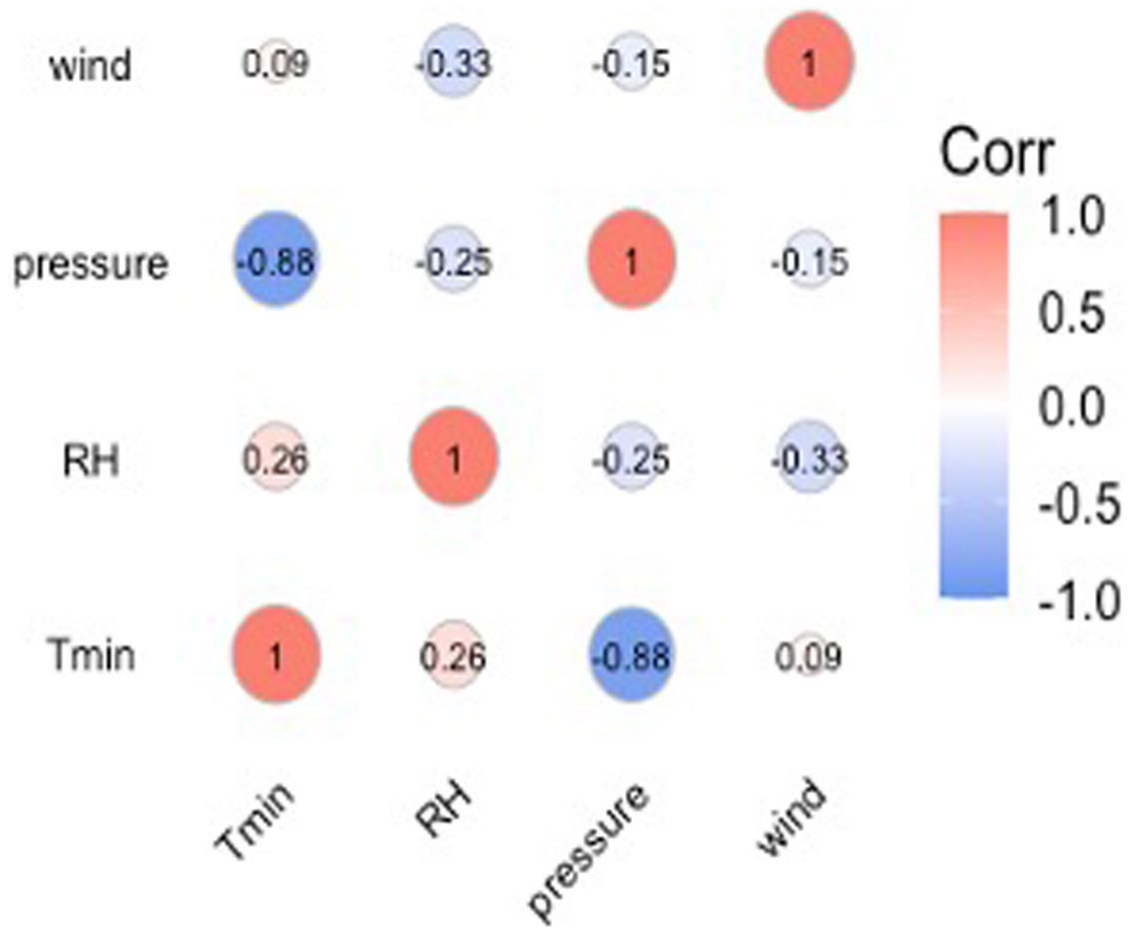
Spearman correlation analysis of meteorological variables.

Figure 2 shows the results of the nonparametric Spearman correlation analysis between 4 meteorological variables (*T*_min_, RH, wind, and pressure). All *p*-values are less than 0.001. Among variables with strong correlations (|r| > 0.7), we kept only one and incorporated it into the final model. In this study, we defined cold waves according to the daily minimum temperature so that there was consistency between cold waves and *T*_min_, so we did not include *T*_min_ in the main model. In addition, there is a strong negative correlation between daily minimum temperature and daily average air pressure, so we only included average wind speed and relative humidity in the final model.

**Figure 3:**
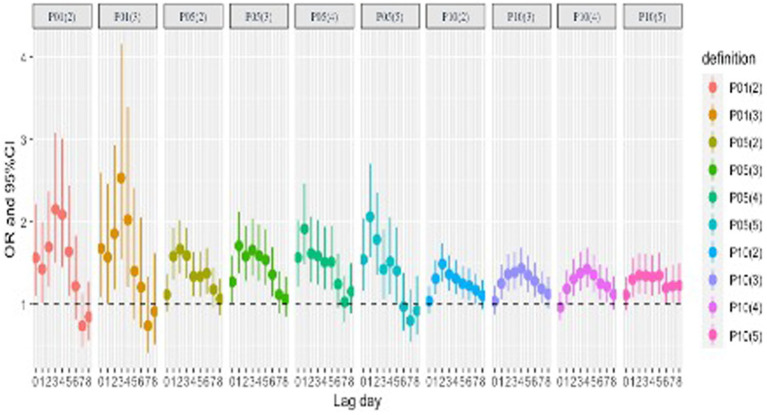
Single-day lag (Lag0-Lag8) results of cold waves and CO events in Jinan from 2013 to 2020.

Figure 3 and Supplementary material S2 show the association between 10 defined cold waves and CO poisoning events under a single-lag model in Jinan. Among the 10 definitions of a cold wave, the day of the cold wave (Lag0) did not have the greatest impact on CO poisoning, and all OR values first increased and then decreased. Under most definitions, cold waves have a significant effect on CO poisoning events, with relatively long lag times (Lag0–Lag5). The effect of the cold wave was significant on the current day (Lag0) with the threshold at P01 or P05 [except for P05(2)]. The largest effects (which indicates that the risk of CO poisoning on a cold wave day is multiple times that on a non-cold wave day under a certain cold wave definition.) of P01(2) and P01(3) appeared in Lag3, 2.15 (95%CI: 1.50–3.08) and 2.53 (95%CI: 1.54–4.16), respectively. The largest effects of P05(2), P05(3), P05(4), and P05(5) appeared in Lag2 (OR = 1.67; 95%CI: 1.37–2.02), Lag1 (OR = 1.71; 95%CI: 1.37–2.12), Lag1 (OR = 1.91; 95%CI: 1.48–2.47), and Lag1 (OR = 2.06; 95%CI: 1.57–2.71). The largest effects of P10(2), P10(3), P10(4), and P10(5) appeared in Lag2 (OR = 1.49; 95%CI: 1.27–1.74), Lag4 (OR = 1.43; 95%CI: 1.22–1.69), Lag4 (OR = 1.42; 95%CI: 1.20–1.69), and Lag2 (OR = 1.35; 95%CI: 1.13–1.62).

**Figure 4:**
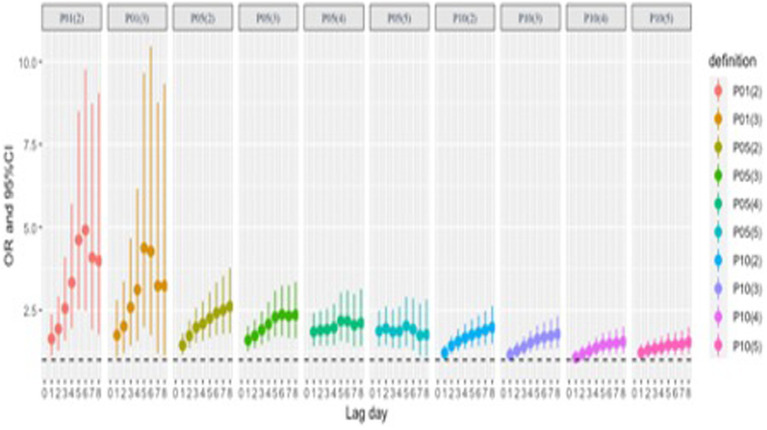
Cumulative impact of cold waves on CO poisoning events in Jinan from 2013 to 2020 (Lag01-Lag08).

Figure 4 and Supplementary material S3 show the cumulative effect of 10 cold wave definitions on CO poisoning events, and these cumulative effects are all statistically significant. As for the trend from Lag01 to Lag08, when the daily minimum temperature P01 was used as the cold wave threshold, the OR of the cumulative effect first increased and then decreased. When P05 and P10 were cold wave thresholds, OR values showed an upward trend before stabilizing.

### 3.1. Sensitivity analysis

**Figure 5:**
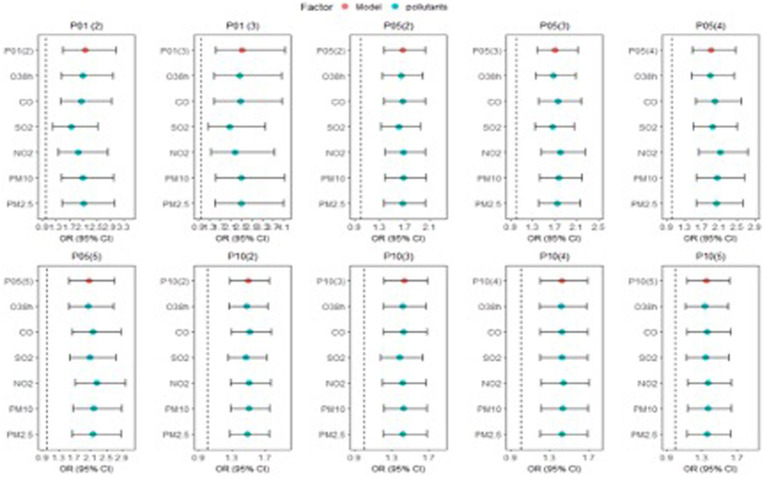
The pollutants were included in the main model to explore the effects of 10 cold wave definitions on CO poisoning in Jinan City.

The results of the sensitivity analysis are shown in Figure 5 and Supplementary material S4. The results are nearly identical before and after adjusting atmospheric pollutants (including PM2.5, PM10, SO_2_, NO_2_, CO, and O_3_), indicating that our findings are stable.

## 4. Discussion

In this study, we systematically examined the effect of cold waves on the incidence of CO poisoning. The CO poisoning events in Jinan have obvious annual cycle characteristics, and the high incidence period is the heating period in winter and spring (November to March of the second year). A key finding of the study was that there was a significant correlation between cold waves and the risk of CO poisoning, and we also observed that the lag effect peaked at days 1–4. Differences in the impact of different definitions of a cold wave were observed, filling a gap in previous research and suggesting the need for specific forecasting systems.

We observed a significant decrease in the number of CO poisoning cases in 2018, which may be due to the fact that the relevant departments of China issued the Clean Winter Heating Plan in Northern China (2017–2021) and issued the overall plan for the gas source guarantee of clean winter heating in key northern regions by replacing coal with gas at the end of 2017. Later, in 2018, Jinan issued its winter clean heating plan (2018–2022), which may have played an effective role in preventing and controlling CO poisoning. In future studies, breakpoint regression and other methods can be used to further study the impact of policy release on CO poisoning.

Our study shows a higher incidence of CO poisoning in winter, which is similar to previous studies. For example, the study by Wang et al. in Taiwan noted that more than 70% of CO poisoning incidents occur in spring and winter (33). A five-year study of Iran by Mitra Rahimi also supports this finding (34). The possible reasons may be as follows. First, when a cold wave hits, it causes the temperature to drop sharply, and it is often accompanied by strong winds and rain or snow (35). People are more inclined to close doors and windows for heating purposes. Second, the greatest impact of the cold wave on CO poisoning events is not on the day of the cold wave but on the days after the cold wave. The reason may be that the heating starts on the day of the cold wave, and the indoor CO concentration does not reach a level that can cause human poisoning that day. After a period of time, the indoor CO level is elevated, leading to poisoning. Third, cold waves are often accompanied by high humidity and high pressure outdoors (36), and it is difficult to discharge CO gas in the house, resulting in gas accumulation. Therefore, in future research, it is necessary to continue to explore the relationship between meteorological factors (such as cold waves) and CO poisoning, clarify the related conditions of CO poisoning, and improve detection and early warning of CO poisoning so that further preventive measures can be taken.

In addition to the above three possible reasons, a more important reason for this phenomenon may be suicidal behavior. Studies have shown an association between ambient temperature and suicidal behavior, and although most studies suggest that high temperatures are more likely to increase suicidal behavior, it goes without saying that suicidal behavior is present at all temperatures (37–39). Therefore, the relevant departments should educate the public on health, pay attention to the public’s mental health, provide psychological counseling to potentially suicidal people, and minimize suicidal behavior, which is particularly important for family happiness and social stability.

In previous studies, there is no unified and clear definition of a cold wave, and most studies have used a temperature threshold and a duration to define a cold wave for research (28, 30), but this single and overly strict definition may underestimate the impact of cold waves on health. However, one definition of a cold wave does not necessarily apply to all regions (40). Furthermore, CO poisoning is a life-threatening emergency (41), and accurate grading of cold waves is critical for assessing cold wave impact and developing cold wave early warning platforms. Therefore, according to the actual situation in Jinan, we used the three percentiles of the daily minimum temperature as the three temperature thresholds, combined with the four durations, and defined 10 cold waves for discussion. We found a strong correlation between CO poisoning events and cold waves. In most cases, the first few days after a cold wave have a major impact on CO poisoning. As the lower temperature threshold decreased and the duration of the cold wave increased, the effect of the cold wave on CO poisoning increased accordingly. This discovery will help the early warning platform to issue earlier, more comprehensive, and accurate warnings in advance. It is helpful to attract people’s attention before the cold wave strikes, ensure efficiency in the corresponding response work, minimize socio-economic and health losses, and avoid wasting health resources.

Jinan City is particularly vulnerable to the dry, cold air mass from Siberia in the winter, resulting in a significant drop in temperature (42). Therefore, it is necessary to take appropriate preventive measures against the onset of cold waves. Our research has proved that cold waves have a significant impact on CO poisoning, so CO poisoning should be highly prioritized by emergency management departments and experts. Relevant departments should propose corresponding policies for the prevention of CO poisoning. First of all, it is necessary to make full use of meteorological data to further improve the early warning ability of CO poisoning. Second, media tools such as radio, television, and the Internet should be used for popular publicity, issuing warnings, and providing education in the critical period before the temperature reaches the trough, effectively increasing the public’s awareness of prevention and self-help and mutual assistance capabilities. Finally, CO detectors should be properly installed and regularly monitored in each home to reduce the occurrence of CO poisoning.

This study has several advantages. First, to our knowledge, this is the first study to set multiple definitions of a cold wave, a time-stratified case-crossover design was also used to explore the association between CO poisoning and cold waves. It combines logistic regression models to control for temporal trends and most confounding factors that do not change in the short term, including economic status, lifestyle, and even genetics. Second, our weather data came from the National Meteorological Information Center of China, and the case data came from the Jinan Emergency Call Center. The authoritativeness of our weather and case data not only reduces bias but also increases the credibility of the findings.

Our study also has some limitations. First, the data on CO poisoning events have some limitations. We were unable to include cases that sought medical attention through other channels and those who died before calling for help, which reduced the statistical power of the study. Second, this study cannot determine the ambient temperature of a specific case, which may lead to biased results. Third, we did not perform subgroup analyses for factors such as age and gender, so the groups of people who are more likely to develop CO poisoning could not be identified. Fourth, as mentioned above, we cannot exclude suicidal behavior, which may bias our results. Therefore, in future studies, we will conduct subgroup analysis according to the intensity of cold waves and case gender and age groups to accurately identify sensitive populations.

## 5. Conclusion

Our study found that exposure to cold waves was associated with an increased risk of CO poisoning events. The magnitude of the effect varies with the temperature threshold and duration of the cold wave; the risk of CO poisoning increases as the threshold decreases and the duration increases. It is necessary to popularize relevant measures to prevent CO poisoning in the public. It is suggested that a cold wave early warning platform be established so that appropriate CO poisoning warnings can be issued based on the severity and characteristics of the cold wave.

## Data availability statement

The original contributions presented in the study are included in the article/Supplementary material, further inquiries can be directed to the corresponding authors.

## Author contributions

JW and LC conceived and designed the study, LC, AR, and YZ directed data analysis and writing. JW and YY analyzed the data. YM, JD, and NC provided writing assistance. JW wrote the manuscript. CZ reviewed the manuscript. All authors contributed to the article and approved the submitted version.

## Funding

This work was supported by the National Science Foundation of China (grant numbers 71974117 and 71774104) and Shandong University Distinguished Young Scholars, the Science and Technology Innovation Development Plan of Jinan City (Clinical Medicine; grant number 202134008).

## Conflict of interest

The authors declare that the research was conducted in the absence of any commercial or financial relationships that could be construed as a potential conflict of interest.

## Publisher’s note

All claims expressed in this article are solely those of the authors and do not necessarily represent those of their affiliated organizations, or those of the publisher, the editors and the reviewers. Any product that may be evaluated in this article, or claim that may be made by its manufacturer, is not guaranteed or endorsed by the publisher.

## Footnotes

^1^ http://www.chinajsb.cn/html/202206/23/27720.html

^2^ http://data.cma.gov.cn/
