# Supplementary material for: Quantifying the effects of cold waves on carbon monoxide poisoning: A time-stratified case-crossover study in Jinan, China
Source: Front Public Health. 2023 Apr 18;11:1050256. doi: 10.3389/fpubh.2023.1050256 (PMC10152301; doi:10.3389/fpubh.2023.1050256)
Supplement: Supplementary file 1 [file Table_1.docx]

**S1. shows the monthly occurrence of CO poisoning events from 2013 to 2020.**

|  | 2013 | 2014 | 2015 | 2016 | 2017 | 2018 | 2019 | 2020 | Sum | percentage（%） |
| --- | --- | --- | --- | --- | --- | --- | --- | --- | --- | --- |
| Jan | 97 | 47 | 49 | 47 | 45 | 60 | 18 | 18 | 381 | 27.49 |
| Feb | 45 | 41 | 38 | 34 | 35 | 11 | 10 | 9 | 223 | 16.09 |
| Mar | 31 | 27 | 26 | 24 | 16 | 6 | 12 | 4 | 146 | 10.53 |
| Apr | 10 | 4 | 7 | 4 | 11 | 0 | 4 | 2 | 42 | 3.03 |
| May | 4 | 3 | 5 | 2 | 8 | 2 | 4 | 4 | 32 | 2.31 |
| Jun | 3 | 2 | 6 | 0 | 1 | 2 | 2 | 3 | 19 | 1.37 |
| Jul | 5 | 2 | 6 | 5 | 4 | 5 | 2 | 3 | 32 | 2.31 |
| Aug | 1 | 2 | 6 | 5 | 10 | 0 | 1 | 4 | 29 | 2.09 |
| Sep | 4 | 4 | 2 | 5 | 1 | 0 | 0 | 1 | 17 | 1.23 |
| Oct | 6 | 7 | 3 | 4 | 2 | 3 | 2 | 1 | 28 | 2.02 |
| Nov | 15 | 30 | 49 | 29 | 20 | 7 | 6 | 8 | 164 | 11.83 |
| Dec | 44 | 57 | 57 | 24 | 36 | 25 | 18 | 12 | 273 | 19.70 |

**S2. Single-day lag (Lag0-Lag8) results of cold waves and CO events in Jinan from 2013 to 2020.**

| definition | Lag0 | Lag1 | Lag2 | Lag3 | Lag4 | Lag5 | Lag6 | Lag7 | Lag8 |
| --- | --- | --- | --- | --- | --- | --- | --- | --- | --- |
| P01(2) | 1.56(1.1,2.21) | 1.43(1.02,2) | 1.69(1.21,2.37) | 2.15(1.5,3.08) | 2.09(1.45,3.01) | 1.64(1.1,2.44) | 1.22(0.81,1.83) | 0.74(0.48,1.13) | 0.84(0.56,1.27) |
| P01(3) | 1.68(1.08,2.6) | 1.57(1,2.46) | 1.86(1.17,2.93) | 2.53(1.54,4.16) | 2.02(1.21,3.39) | 1.4(0.81,2.41) | 1.21(0.71,2.06) | 0.74(0.41,1.33) | 0.91(0.51,1.62) |
| P05(2) | 1.11(0.91,1.37) | 1.58(1.3,1.93) | 1.67(1.37,2.02) | 1.59(1.31,1.93) | 1.34(1.09,1.63) | 1.34(1.09,1.64) | 1.37(1.12,1.68) | 1.18(0.96,1.44) | 1.06(0.87,1.3) |
| P05(3) | 1.27(1.02,1.59) | 1.71(1.37,2.12) | 1.58(1.28,1.95) | 1.66(1.34,2.04) | 1.59(1.28,1.97) | 1.54(1.24,1.91) | 1.36(1.09,1.69) | 1.11(0.89,1.4) | 1.06(0.85,1.33) |
| P05(4) | 1.56(1.21,2.02) | 1.91(1.48,2.47) | 1.61(1.26,2.06) | 1.58(1.24,2.02) | 1.51(1.17,1.94) | 1.51(1.17,1.95) | 1.24(0.95,1.61) | 1.02(0.78,1.34) | 1.15(0.89,1.5) |
| P05(5) | 1.54(1.16,2.04) | 2.06(1.57,2.71) | 1.78(1.35,2.35) | 1.42(1.06,1.91) | 1.51(1.12,2.05) | 1.4(1.02,1.93) | 0.97(0.68,1.38) | 0.8(0.54,1.18) | 0.92(0.63,1.34) |
| P10(2) | 1.04(0.88,1.22) | 1.31(1.12,1.53) | 1.49(1.27,1.74) | 1.36(1.16,1.6) | 1.31(1.12,1.54) | 1.25(1.06,1.46) | 1.22(1.05,1.43) | 1.17(1,1.37) | 1.1(0.94,1.29) |
| P10(3) | 1.03(0.87,1.22) | 1.25(1.06,1.48) | 1.36(1.16,1.61) | 1.38(1.17,1.63) | 1.43(1.22,1.69) | 1.35(1.15,1.59) | 1.28(1.09,1.51) | 1.18(1,1.4) | 1.12(0.95,1.34) |
| P10(4) | 0.96(0.8,1.15) | 1.19(0.99,1.41) | 1.31(1.1,1.55) | 1.38(1.16,1.63) | 1.42(1.2,1.69) | 1.35(1.14,1.6) | 1.25(1.05,1.48) | 1.21(1.02,1.44) | 1.12(0.93,1.34) |
| P10(5) | 1.11(0.92,1.33) | 1.3(1.08,1.56) | 1.35(1.13,1.62) | 1.34(1.12,1.61) | 1.33(1.1,1.6) | 1.35(1.12,1.62) | 1.19(0.99,1.44) | 1.22(1.01,1.48) | 1.23(1.01,1.49) |

**S3. Cumulative impact of cold waves on CO poisoning events in Jinan from 2013 to 2020 (Lag01-Lag08**

| definition | Lag01 | Lag02 | Lag03 | Lag04 | Lag05 | Lag06 | Lag07 | Lag08 |
| --- | --- | --- | --- | --- | --- | --- | --- | --- |
| P01(2) | 1.63（1.12,2.39） | 1.93（1.28,2.93） | 2.55（1.59,4.11） | 3.33（1.94,5.7） | 4.62（2.51,8.5） | 4.92（2.48,9.76） | 4.09（1.91,8.74） | 3.98（1.76,9.05） |
| P01(3) | 1.75（1.09,2.81） | 2.01（1.2,3.37） | 2.58（1.43,4.67） | 3.12（1.58,6.16） | 4.37（1.98,9.66） | 4.27（1.74,10.46） | 3.23（1.19,8.77） | 3.23（1.12,9.33） |
| P05(2) | 1.44（1.15,1.81） | 1.73（1.36,2.2） | 1.98（1.52,2.57） | 2.09（1.58,2.77） | 2.25（1.67,3.05） | 2.42（1.75,3.33） | 2.51（1.78,3.54） | 2.61（1.81,3.78） |
| P05(3) | 1.6（1.26,2.02） | 1.73（1.35,2.21） | 1.9（1.46,2.47） | 2.08（1.57,2.75） | 2.29（1.7,3.08） | 2.36（1.72,3.24） | 2.33（1.66,3.25） | 2.36（1.66,3.35） |
| P05(4) | 1.85（1.41,2.42） | 1.89（1.43,2.5） | 1.91（1.43,2.56） | 1.97（1.45,2.69） | 2.18（1.57,3.03） | 2.17（1.52,3.09） | 2.05（1.41,2.99） | 2.11（1.42,3.14） |
| P05(5) | 1.87（1.4,2.49） | 1.95（1.45,2.62） | 1.85（1.36,2.53） | 1.87（1.34,2.61） | 2.03（1.41,2.91） | 1.92（1.29,2.86） | 1.74（1.12,2.7） | 1.76（1.1,2.82） |
| P10(2) | 1.21（1.01,1.45） | 1.42（1.17,1.73） | 1.56（1.26,1.93） | 1.67（1.33,2.09） | 1.75（1.38,2.22） | 1.83（1.42,2.35） | 1.9（1.46,2.48） | 1.98（1.49,2.63） |
| P10(3) | 1.16（0.97,1.39） | 1.28（1.06,1.55） | 1.39（1.13,1.7） | 1.52（1.22,1.88） | 1.62（1.29,2.04） | 1.68（1.33,2.14） | 1.73（1.34,2.22） | 1.78（1.36,2.33） |
| P10(4) | 1.07（0.89,1.29） | 1.18（0.97,1.44） | 1.27（1.04,1.56） | 1.37（1.1,1.69） | 1.45（1.16,1.81） | 1.48（1.18,1.87） | 1.51（1.18,1.93） | 1.55（1.2,2） |
| P10(5) | 1.22（1,1.48） | 1.3（1.07,1.59） | 1.34（1.09,1.65） | 1.38（1.11,1.71） | 1.45（1.16,1.81） | 1.45（1.15,1.84） | 1.47（1.15,1.89） | 1.53（1.18,2） |

**S4. The pollutants were included in the main model to explore the effects of 10 cold wave definitions on CO poisoning in Jinan City.**

| definition | | Main model | +PM2.5 | +PM10 | +SO2 | +NO2 | +CO | +O3 |
| --- | --- | --- | --- | --- | --- | --- | --- | --- |
| P01(2) | 2.15(1.5,3.08) | | 2.11(1.46,3.03) | 2.1(1.46,3.01) | 1.74(1.19,2.54) | 1.96(1.36,2.82) | 2.05(1.43,2.94) | 2.08(1.45,2.99) |
| P01(3) | 2.53(1.54,4.16) | | 2.5(1.52,4.11) | 2.51(1.53,4.12) | 2.05(1.24,3.41) | 2.27(1.38,3.75) | 2.46(1.49,4.04) | 2.43(1.48,4) |
| P05(2) | 1.67(1.37,2.02) | | 1.66(1.37,2.02) | 1.67(1.38,2.03) | 1.61(1.32,1.95) | 1.67(1.38,2.03) | 1.66(1.37,2.02) | 1.64(1.35,1.99) |
| P05(3) | 1.71(1.37,2.12) | | 1.75(1.4,2.17) | 1.77(1.42,2.2) | 1.66(1.33,2.06) | 1.81(1.45,2.25) | 1.76(1.41,2.18) | 1.68(1.35,2.08) |
| P05(4) | 1.91(1.48,2.47) | | 2.03(1.56,2.63) | 2.05(1.58,2.66) | 1.94(1.5,2.51) | 2.12(1.63,2.75) | 2(1.54,2.59) | 1.89(1.46,2.44) |
| P05(5) | 2.06(1.57,2.71) | | 2.16(1.64,2.85) | 2.18(1.65,2.88) | 2.08(1.58,2.74) | 2.25(1.71,2.98) | 2.17(1.65,2.85) | 2.04(1.56,2.68) |
| P10(2) | 1.49(1.27,1.74) | | 1.49(1.27,1.74) | 1.5(1.28,1.75) | 1.46(1.25,1.71) | 1.5(1.28,1.76) | 1.51(1.29,1.77) | 1.47(1.26,1.72) |
| P10(3) | 1.43(1.22,1.69) | | 1.42(1.21,1.67) | 1.43(1.22,1.68) | 1.39(1.18,1.64) | 1.42(1.2,1.67) | 1.42(1.21,1.67) | 1.42(1.21,1.67) |
| P10(4) | 1.42(1.2,1.69) | | 1.42(1.2,1.68) | 1.43(1.21,1.69) | 1.43(1.21,1.69) | 1.44(1.21,1.7) | 1.42(1.2,1.68) | 1.41(1.19,1.68) |
| P10(5) | 1.35(1.13,1.62) | | 1.35(1.13,1.62) | 1.36(1.14,1.64) | 1.34(1.12,1.61) | 1.36(1.14,1.63) | 1.35(1.13,1.62) | 1.33(1.11,1.6) |
